# Optimal movement behaviors: correlates and associations with anxiety symptoms among Chinese university students

**DOI:** 10.1186/s12889-021-12116-6

**Published:** 2021-11-09

**Authors:** He Bu, Ai He, Na Gong, Liuyue Huang, Kaixin Liang, Kaja Kastelic, Jiani Ma, Yang Liu, Si-Tong Chen, Xinli Chi

**Affiliations:** 1 Department of Social and Behavioural Sciences, City University of Hong Kong, Hong Kong, SAR China; 2 School of Sport Medicine and Physical Therapy, Beijing Sport University, Beijing, China; 3 School of Psychology, Shenzhen University, Shenzhen, China; 4 Andrej Marusic Institute, University of Primorska, Koper, Slovenia; 5 Human Health in the Built Environment, InnoRenew CoE, Izola, Slovenia; 6 Centre for Sport, Exercise and Health Sciences, Coventry University, Coventry, UK; 7 School of Health and Social Development, Deakin University, Geelong, Australia; 8 School of Physical Education and Sport Training, Shanghai University of Sport, Shanghai, China; 9 Shanghai Research Centre for Physical Fitness and Health of Children and Adolescents, Shanghai University of Sport, Shanghai, China; 10 Institute for Health and Sport, Victoria University, Melbourne, Australia

**Keywords:** Anxiety, Anxiety symptoms, University students, Physical activity, Sedentary behavior, Sleep

## Abstract

**Background:**

The Canadian 24-Hour Movement Guidelines for Adults was released in 2020. There is a dearth of evidence on the association between adherence to the 24 h movement guidelines and health indicators. This study aims to (a) explore the associations between potential correlates and meeting the 24 h movement guidelines using a sample of Chinese university students; and (b) examine if meeting 24 h movement guidelines is associated with the severity of anxiety symptoms.

**Methods:**

Cross-sectional findings are based on 1846 Chinese university students (mean age = 20.7 years, 64.0% female). Movement behaviors (physical activity, sedentary behavior, and sleep duration), possible correlates, and anxiety symptoms were measured through self-reported online questionnaires. Logistic regression models were performed to examine the associations.

**Results:**

We found that male students and those who had a mother with a master’s degree or above, more close friends and higher perceived family affluence were more likely to meet the overall 24 h guidelines. Meeting all 24 h movement guidelines presented the lower odds for severe anxiety symptoms than those meeting fewer recommendations in the 24 h movement guidelines.

**Conclusions:**

As one of the first to examine the correlates of adherence to the 24 h movement guidelines and the relationship between anxiety symptoms and meeting the guidelines among Chinese university students, our findings contribute to the growing body of evidence linking movement behaviors, psychosocial correlates, and heath indicators. Schools and health providers can encourage movement behaviors that follow the guidelines on campus.

## Introduction

In the transition from adolescence to adulthood, university students are often exposed to heavy psychosocial pressures, making them more vulnerable to mental health problems [1, 2]. Data has shown that anxiety, characterized by excessive fears or worries [3, 4], has emerged as one of the most prevalent and severe mental health problems among Chinese university students, with a prevalence rate of anxiety symptoms from 7.6 to 54.4% among Chinese university students (age 15–29) in the past decade [5–10]. Anxiety symptoms are linked to various adverse life outcomes, including lower educational attainment, reduced employment, and poor quality of life [11]. Previous cross-cultural studies revealed that Chinese university students tended to be more anxious than their American counterparts [12]. This finding addressed the necessity of investigating the anxiety symptoms and identifying their unique correlates among Chinese university students, which can inform appropriate preventive intervention against anxiety and further facilitate an all-around development of Chinese university students.

### 24 h movement behaviors

From a perspective of movement behavior, sleep, sedentary behavior (SB), and physical activity (PA) are beneficial to mental health outcomes. Extensive literature has documented significant associations between anxiety symptoms and sleep [13], PA [14], and SB [15]. For example, in the sample of Chinese university students, negative associations were found between PA and anxiety symptoms [16, 17]. Liu et al., observed the bidirectional longitudinal relationships between SB (e.g., long-time mobile phone use) and the severity of anxiety symptoms among Chinese university students [18]. Among Chinese students (aged 18–25), anxiety symptoms are significantly associated with sleep duration [19]. Although traditional studies highlighted the independent effect of the single movement behavior and confirmed the independent roles, those studies had a poor adjustment for time spent in other movement behaviors [20], which overlooks a clear fact that individual has a fixed budget of 24 h a day that was entirely composed of PA, SB, and sleep. In other words, any shift in time invested in a single one of these movement behaviors could inevitably increase or decrease the remaining [21]. Consequently, it is necessary to emphasize that “the whole day matters” and investigate the optimal balance among the time spent in 24 h movement behaviors and the combined health benefits of these co-dependent and interrelated behaviors [20–22].

This holistic perspective of 24 h movement behavior is in line with the integrated vs. isolated movement behavior paradigm and the theoretical framework of studying health-related time-use patterns, *Framework for Viable Integrative Research in Time-Use Epidemiology* (VIRTUE), which guides studies on methods, determinants, composition, outcomes, and interventions of optimally integrated patterns of time-use for population health [20, 22]. With this lens, *Canadian 24-Hour Movement Guidelines for Adults aged 18–64 years*, a set of health recommendations of the combination of PA, SB, and sleep, was issued [23, 24]. The fundamental principle of 24 h movement guidelines is to shift the focus from a single movement behavior recommendation to an integrated guideline that contains combined recommendations for PA, SB and sleep throughout the whole day [15, 23]. A recent systematic review examining associations between the 24 h movement compositions and health outcomes reinforces the rationale for packaging recommendations for PA, SB, and sleep into an integrated guideline [25]. Specifically, it is recommended that for an adults to be considered to have a healthy movement behavior profile, they need to meet three specific recommendations for time-use of PA (accumulated ≥150 min of moderate-to-vigorous PA per week), SB (≤ 8 h per day) and sleep (7–9 h per day) [23]. A growing body of evidence has documented these guidelines’ value, demonstrating that the combined effect of 24 h movement behavior throughout the day is vital to various health outcomes for children and youth [26, 27] and may have important implications for health across the adult lifespan [22]. To the best of our knowledge, although the 24 h movement guidelines for adults has been issued, there is a dearth of evidence on the association between adherence to the 24 h movement guidelines and anxiety symptoms. Therefore, we aimed to explore the associations between meeting 24 h movement guidelines and the severity of anxiety symptoms.

Moreover, according to the behavioral epidemiology framework [28], identifying factors is also fundamental to determine whether particular behaviors should be targeted in health interventions. Many factors (e.g., sociodemographic, lifestyle, and environmental factors) would influence the development and adherence to movement guidelines [22, 29]. For example, it was shown previously that female students from the Chinese campus have a significantly higher prevalence of low PA, long sedentary time, and poor sleep quality [17, 30]. Short sleepers tend to have low income and be overweight [31]. A study on university students from 23 low-, middle- and high-income countries across the world has revealed that in 17 out of 23 countries, including China, being female is associated with physical inactivity [32]. Additionally, older age (22–30 years), studying in a low- or lower-middle-income country, unhealthy weight status (i.e., underweight, overweight or obese), and lack of social support are also associated with physical inactivity [32].

### The present study

Movement behaviors may have the potential to benefit the mental health of university students. With a holistic lens of 24 h movement behaviors, the current study followed specific recommendations of Canadian 24-Hour Movement Guidelines for Adults aged 18–64 years for time-use of PA, SB, and sleep, and aimed to: [1] explore the associations between potential correlates and meeting the 24 h movement guidelines using a sample of Chinese university students; [2] investigate the association between meeting 24 h movement guidelines and anxiety symptoms.

## Methods

### Participants

This study employed a cross-sectional design using online surveys. A convenient sampling strategy was adopted to recruit participants. A total of 1942 university students were recruited from 30 provinces and autonomous regions (mainly from Guangdong province), with 1846 participants (response rate = 95.1%) providing answers to all of the study’s variables. Before data was collected, study participants provided their consent to participate in the research through an online survey.

### Procedure and data collection

Data collection was conducted from August 21 to 31, 2020. At that time, COVID-19 had been controlled in China, and college students were about to return to campus. Participants completed the survey through online platforms (e.g., Tencent’s QQ, Tencent’s WeChat, Sina Weibo, and campus forums) due to COVID-19 restrictions. It took approximately 15 min for each participant to complete the online questionnaire, and each one received compensation for their time spent on the survey (10 CNY, equivalent to 1.5 USD) via online payment for their participation.

### Measures

#### Movement behaviors

Movement behaviors including PA, SB, and sleep were assessed via two self-report questionnaires, including the International Physical Activity Questionnaire-Short Form (IPAQ-SF) and Pittsburgh Sleep Quality Index (PSQI). The good psychometric properties of the IPAQ-SF and PSQI have been shown in Chinese populations [33, 34].

For PA, six items of IPAQ-SF were adopted. Participants were required to recall the frequency and duration of 1) walking activities; 2) moderate PA (e.g., carrying light loads, bicycling at a regular pace, or doubles tennis); and 3) vigorous PA (e.g., heavy lifting, digging, aerobics, or fast bicycling) that they spent over the past seven days. Example item: *During the last seven days, on how many days did you do vigorous physical activities like heavy lifting, digging, aerobics, or fast bicycling?* Based on the Canadian 24-Hour Movement Guidelines for Adults [23], participants that accumulated ≥150 min of moderate-to-vigorous PA per week were categorized as meeting the 24 h movement guidelines.

For SB, one item of IPAQ-SF was adopted: *During the last seven days, how much time did you spend sitting on a weekday? Include time spent at work, at home, while doing course work and during leisure time. This may include time spent sitting at a desk, visiting friends, reading, or sitting or lying down to watch television.* Meeting the Canadian 24-Hour Movement Guidelines for Adults requires ≤8 h of SB per day [23].

For sleep, one item from the PSQI was used to assess the sleep duration: *During the past month, how many hours of actual sleep did you get at night? (This may be different from the number of hours you spent in bed).* Meeting the Canadian 24-Hour Movement Guidelines for Adults requires 7–9 h of sleep daily [23].

Participants were classified into four groups based on whether they met all 24 h movement guidelines, any combination of two individual guidelines, any single individual guideline, or none of the guidelines. Synthetization as numbers can directly examine participants’ movement behavior profiles and their associated mental health outcomes. This approach can help test whether meeting more movement behavior recommendations is associated with better health outcomes.

#### Anxiety symptoms

The Chinese version of Zung’s Self-rating Anxiety Scale (SAS) has been employed to assess participants’ anxiety symptoms, which has been widely applied in recent research on university students in the context of COVID-19 [35–37]. SAS consists of 20 items, and each item is rated on a scale from 1 to 4. The total raw score is multiplied by 1.25 and then converted into a standardized index score ranging between 25 and 100, with higher scores suggesting more severe anxiety. SAS has shown the capacity to discriminate between clinical and non-clinical samples [38]. The norms that developed for the Chinese population include an index score of 50 as the cut-off point of clinical significance and index scores of symptom severity (i.e., index score 50–60, 61–70, > 70 indicating mild, moderate, severe anxiety, respectively) [39]. These norms have been widely used among the Chinese population as a valuable screening tool for the presence of anxiety symptoms [39]. Therefore, In the current study, SAS ≥ 50 was applied as the cut-off point beyond which scores are considered indicative of the presence of anxiety symptoms.

#### Potential correlates

Based on previous studies [40], potential correlates included body mass index (BMI; the body weight divided by the square of the body height [kg/m^2^]), age (years), sex (male or female), siblings (single, two or more), residence (urban or rural), family structure (complete, divorced or other), parents’ educational level (middle school or below, high school, college or university, master or above), number of friends (none, 1–2, 3–5, 6 or more), and perceived family affluence using a scale (from 0 to 10) with higher scores indicating higher perceived family affluence [41].

### Statistical analyses

Statistical analyses were performed using STATA 16.1 (Stata Corp, College Station, Texas). Descriptive statistics were used to report the frequency (percentage) and mean (standard deviation [SD]) of categorical and continuous variables, respectively.

To explore the associations between sociodemographic correlates and meeting the 24 h movement guidelines, we performed a binary logistic regression model to examine if meeting the 24 h movement guidelines was associated with the potential correlates. Whether meeting the 24 h movement guidelines (i.e., meeting none of the guidelines vs. meeting at least one of the guidelines) was treated as the dependent variable, and potential correlates were treated as independent variables. All potential correlates were entered in the regression model at once. Meeting none of the guidelines was set as the reference group. Odds ratios (OR) with 95% confidence interval (CI) were reported. Statistical significance was set up as *p* < 0.05 (two-sided).

To examine the relationship between meeting 24 h movement guidelines and anxiety symptoms (SAS ≥ 50), we used a binary logistic regression model, with meeting 24 h movement guidelines (i.e., number of recommendations met) as the independent variable and anxiety symptoms (i.e., SAS ≥ 50 vs. SAS < 50) as the dependent variable. Correlates including sex, age, siblings, residence, family structure, father education level, mother education level, perceived family affluence, number of friends, and body mass index were controlled. Odds ratios (OR) with 95% confidence interval (CI) were reported. Statistical significance was set up as *p* < 0.05 (two-sided).

## Results

### Sample characteristics

As presented in Table 1, the mean age of the sample was 20.7 years (SD = 1.6), and the average BMI (kg/m^2^) was 20.3 (SD = 2.9). Overall, the prevalence of meeting the 24-Hour movement guidelines was 27.0%. Participants reported a mean score of 41.8 ± 9.8 for anxiety symptoms. The largest proportion of participants were categorized as having minimal anxiety symptoms (79.2%), and 21.8% of participants indicated mild-to-severe anxiety symptoms.

**Table 1:** Descriptive characteristics of study sample (*n* = 1846)

	n (%) / Mean (SD)	
Age (years)	20.7	(1.6)
**BMI (kg/m**^**2**^**)**	20.3	(2.9)
**Sex**		
Male	665	(36.0)
Female	1181	(64.0)
**Siblings**		
Single	639	(34.6)
Two or more	1207	(65.4)
**Residence**		
Urban	1278	(69.2)
Rural	568	(30.8)
**Family structure**		
Full	1664	(90.1)
Divorced	117	(6.3)
Other	65	(3.5)
**Perceived family affluence**	5.7	(1.6)
**Father education level**		
Middle school or below	891	(48.3)
High school	636	(34.5)
College or university	254	(13.8)
Master or above	65	(3.5)
**Mother education level**		
Middle school or below	1086	(58.8)
High school	560	(30.3)
College or university	158	(8.6)
Master or above	42	(2.3)
**Number of friends**		
None	29	(1.6)
1–2	608	(32.9)
3–5	964	(52.2)
6 or more	245	(13.3)
**Meeting the physical activity guidelines**		
No	950	(51.5)
Yes	896	(48.5)
**Meeting the sedentary behavior guidelines**		
No	575	(31.1)
Yes	1271	(68.9)
**Meeting the sleep guidelines**		
No	555	(30.1)
Yes	1291	(69.9)
**Meeting combinations of 24 h movement guidelines**		
None	119	(6.4)
One	495	(26.8)
Two	733	(39.7)
All	499	(27.0)
**Anxiety symptoms (scores: mean ± SD)**	41.8	(9.8)
**Anxiety symptoms (severity)**		
Minimal anxiety (SAS < 50)	1462	(79.2)
Mild anxiety (SAS 50–60)	277	(15.0)
Moderate anxiety (SAS 61–70)	89	(4.8)
Severe anxiety (SAS > 70)	18	(1.0)

Note. SD = standard deviation. SAS = Index score of Zung’s Self-rating Anxiety Scale

### Associations between sociodemographic correlates and meeting the 24 h movement guidelines

Table 2 presents the results of correlates of meeting the 24 h movement guidelines. For intrapersonal correlates (i.e., sex, age, and BMI), sex and BMI showed significant cross-sectional associations with the adherence to 24 h movement guidelines. Specifically, compared with the female, male participants were more likely to meet the guidelines (OR = 2.20, 95% CI [1.75, 2.77]). University students with greater BMI were more likely to meet the guidelines (OR = 1.04, 95% CI [1.00, 1.08]).

**Table 2:** Correlates of meeting the 24 h movement guidelines

	OR	95% CI	
***Intercept***	0.21	0.03	1.32
**Body mass index**	1.04	1.00	1.08
**Age**	0.98	0.91	1.05
**Sex**			
Male	2.20	1.75	2.77
Female	Ref		
**Siblings**			
Single	1.01	0.80	1.29
Two or more	Ref		
**Residence**			
Urban	1.03	0.80	1.33
Rural	Ref		
**Family structure**			
Full	0.90	0.50	1.62
Divorced	0.76	0.37	1.58
Other	Ref		
**Perceived family affluence**	1.13	1.05	1.21
**Father education level**			
Middle school or below	1.28	0.64	2.57
High school	1.39	0.70	2.75
College or university	1.74	0.87	3.48
Master or above	Ref		
**Mother education level**			
Middle school or below	0.42	0.19	0.93
High school	0.42	0.19	0.91
College or university	0.43	0.19	0.97
Master or above	Ref		
**Number of friends**			
None	1.27	0.55	2.93
1–2	0.64	0.45	0.90
3–5	0.97	0.71	1.33
> 6	Ref		

Note. Meeting none of the guidelines was set as the reference group

For interpersonal factors, significant associations were found between mother educational level, perceived family affluence level, numbers of friends, and the likelihood of meeting the guidelines. Specifically, compared with the mother’s educational level of master degree or above, other educational attainment showed a lower likelihood of meeting the guidelines (OR = 0.42–0.43; 95% CI [0.19, 0.91–0.97]). Participants who reported higher perceived family affluence were better compliant with the guidelines (OR = 1.13, 95% CI [1.05, 1.21]). Participants with 1–2 friends were less likely to meet the guidelines than those with six or more friends (OR = 0.64, 95% CI [0.45, 0.90]). No significant association was observed between family structure, the father’s educational level, and the likelihood of meeting the guidelines.

### Associations between meeting the 24 h movement guidelines and anxiety symptoms

**Fig. 1:**
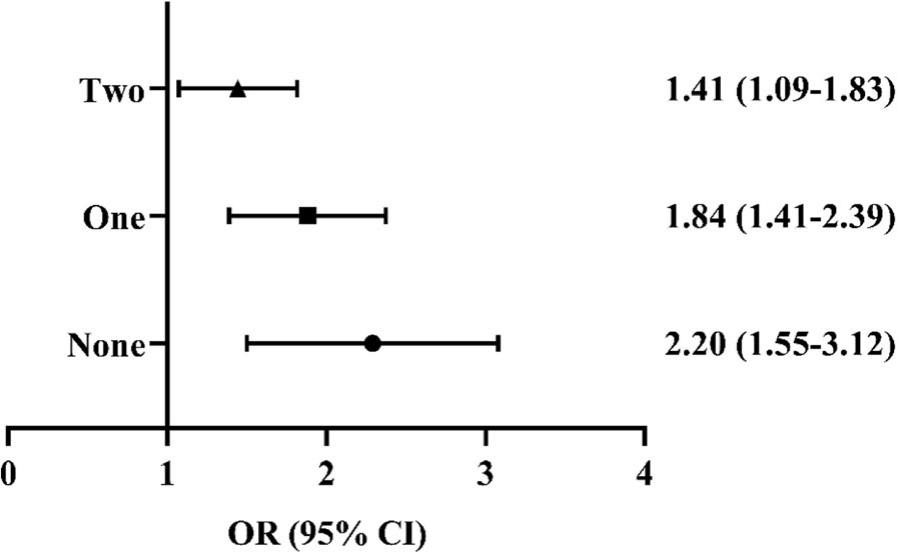
The association between meeting 24 h Guidelines and anxiety symptoms screened positive by Generalized Linear Models. Notes: Models controlled for sex, age, siblings, residence, family structure, father education level, mother education level, perceived family affluence, number of friends, body mass index. Meeting all 24 h movement guidelines was set as the reference group. Two = meeting any two recommendations of the 24 h movement guidelines. One = meeting any one recommendation of the 24 h movement guidelines. None = meeting none of the 24 h movement guidelines

Figure 1 shows the results. Compared with those who met all 24 h movement guidelines (i.e., PA, SB, and sleep recommendation), participants who met only two recommendations were more likely to have anxiety symptoms (OR = 1.41, 95%CI [1.09, 1.83] *p* < 0.001). Likewise, the risk for anxiety symptoms of participants who met only 1 guideline increased to 1.84 times compared with the reference group (OR = 1.84, 95%CI [1.41, 2.39], *p* < 0.001). Finally, the odds of anxiety symptoms among participants following no recommendations was 2.20 times as the reference group that meeting the guidelines (OR = 2.20, 95%CI [1.55, 3.12], *p* < 0.001).

## Discussion

To the authors’ knowledge, the current study is one of the first to investigate the correlates of meeting 24 h movement guidelines and its association with anxiety symptoms in a sample of Chinese university students. Those who were male students, had a mother with a master’s degree or above, had more close friends, and perceived higher family affluence were more likely to meet the integrated 24 h guidelines. Among university students, meeting the 24 h movement guidelines presented lower odds for anxiety symptoms than those meeting fewer recommendations in the 24 h movement guidelines.

Understanding factors of compliance with the guidelines is essential for health promotion efforts [22, 28]. Research on the demographic correlates of meeting the 24 h movement guidelines helps identify university students who are most in need of intervention [28]. These demographic correlates may exert potential moderating effects on associations between adherence to the guidelines and health outcomes [22]. However, upon the release of the Canadian 24-Hour Movement Guidelines for Adults in October 2020, current evidence among Chinese university students is insufficient, which limited the capacity of health professionals to target populations with health risks. In other Asian countries, mixed findings were reported. For example, Korean adults who are male, have low incomes, live in nonmetro areas, and have a lower education background tended to comply with the 24 h movement guidelines [42]. Thailand issued a national 24 h movement behavior guidelines, including the recommendations of PA (i.e., at least 150 min/week of moderate and vigorous PA), sleep (between 7 to 9 h per day), and SB (interrupt SB every two hours) [43]. The higher odds for meeting the Thai guidelines were observed among females, those living in rural areas, employed, and those with high education levels [44]. The variations may be attributed to diverse measurements and sample characteristics in different studies, resulting in discrepant research findings. For example, the Thai study used a 24 h time-use diary to assess movement behaviors, and the sample of Thai and Korean study had a wider age range (Thai: 18–59; Korean: 18–64) compared to our sample aged from 18 to 26 years [42, 44]. The Thai study used Thai guidelines that provided recommendations to interrupt SB every 2 h, not to limit SB time to 8 h or less [42].

When comparing the results with those in Chinese children and adolescents, our study yielded similar results that higher parental education levels and family income may facilitate favorable 24 h movement behaviors [26, 44]. The underlying mechanism may be that, like children and adolescents, most university students have not yet achieved financial independence and are dependent on their families in terms of socioeconomic status [45]. Moreover, households of higher socioeconomic provided more opportunities for adopting healthy lifestyles (e.g., less SB and more PA) [46] and maintaining active lifestyles (e.g., more PA) from childhood to adulthood [47]. However, family socioeconomic status cannot represent the whole picture of family-level correlates. Family functioning also plays a vital role in shaping children’s health behaviors [44, 48, 49]. More studies are needed to further explore the influence of multifaceted factors (e.g., family, school, community, and environment) on the adherence to the 24 h movement guidelines [15, 22].

Moreover, in this study, the higher odds for meeting the guidelines were observed in university students with greater BMI. This finding should be interpreted with caution since the value of the odds ratio of BMI was nearly equal to 1 (OR = 1.04), and the lower bound of 95% CI was at the edge of the threshold for significance (95% CI: 1.00 to 1.08). Recent studies found that the distribution of adults’ time-use in movement behaviors was significantly associated with adiposity indicators (i.e., BMI and waist circumference) [22, 44, 50, 51]. Chinese children and adolescents who engaged in the ideal 24 h movement guidelines were shown to be less prone to develop overweight and obesity [26, 44]. Research comparable to those conducted for children and adolescents could be undertaken in adult populations in China and abroad to explore the associations.

Prior research has shown the significant associations between adhering to the 24 h movement guidelines and health benefits across younger populations [22, 27, 44, 52]. The present study found that meeting all recommendations of the guidelines showed a lower odds ratio for higher anxiety symptoms. Our finding is similar to a recent study conducted in western countries indicating that the probability of health benefits increased with the number of guidelines that the adults followed increased [53]. Some of the core biological mechanisms thought to be related to anxiety symptoms may explain the association between movement behaviors and anxiety symptoms. The visceral-afferent feedback model suggests that physical activity may increase the stimulation of the ascending reticular activating system (ARAS), responsible for arousal and the maintenance of wakefulness [54]. When the cortical excitation from physical activity reaches a point at which the inhibitory mechanism of ARAS is activated, the somatic afferent stimulation ultimately decreases [54, 55]. The currently available first-line treatments for anxiety disorders work by interacting with drugs and inhibitory neurotransmitters [56].

### Strengths and limitations

A clear and evidence-based guideline on 24 h movement behaviors and its associations with health outcomes could help set measurable indicators for surveillance, provide appropriate guidance to public health professionals, and promote healthy lifestyles among the mass public. As one of the first to assess links between movement behaviors, the correlates, and anxiety symptoms among Chinese university students, our findings show the potential of using this guideline in the field of public health in China by enriching the evidence on 24 h movement behavior guidelines. Our findings also informed preventive intervention design. Chinese university students who are female and have low socioeconomic status should be prioritized as the target population for future programs encouraging optimal movement behaviors. Schools and health professionals can facilitate movement behaviors that comply with the recommendations on campus as a cost-effective solution to alleviate anxiety symptoms and promote mental health.

Notably, there may be differences in university students’ optimal movement behaviors across distinct socio-cultural backgrounds. Existing studies indicated that young people from eastern countries reported lower levels of PA [57], higher levels of SB [30], and shorter sleep duration [58, 59] than their western counterparts, especially in eastern Asian countries. This might be because of the sedentary culture induced by the intensive competition of the higher education, wherein college students have to sacrifice PA and sleep time for better academic performance [60]. Instead of precise recommendations for each behavior that adds up to 24 h, the guidelines provide ranges, which to some degree “respect the individuality, variability, and personal preferences of the end-user” across cultures [23]. However, we encourage researchers to re-evaluate the evidence, especially involving evidence generated from their own cultural contexts, to help develop and improve the optimal 24 h movement behavior composition, and not to adopt such recommendations uncritically just for “fear of missing out” [61].

Our results should be interpreted considering methodological limitations. First, due to the study’s cross-sectional nature, we cannot infer the causal relationship between meeting 24 h movement guidelines, its correlates, and anxiety symptoms. Recent research has adopted a longitudinal design to track the adherence to 24 h movement guidelines at multiple time points and detect longitudinal associations between adherence to 24 h movement guidelines and adiposity over time [62]. Additionally, a bidirectional association might exist between the adherence to the 24 h movement guidelines and mental health since recent research has found that university students who reported better mental health seem to be more likely to meet the guidelines [63]. Therefore, more longitudinal studies are warranted to examine the causality.

Second, the accuracy of data and estimation may be affected during the COVID-19 pandemic. Although our data were collected at the time when most cities in China were classified as low-risk areas, the level of movement behaviors might have been affected due to people’s cautious attitude towards going outside and the strict preventive and control measures. Moreover, recall bias and the exclusive use of self-reported data could also influence the accuracy of data. Future studies can apply device-based measures (e.g., pedometers, accelerometers, heart rate monitors) or combine them with subjective ones (e.g., diaries and questionnaires) to assess movement behaviors [64].

Third, our data was collected through convenient sampling on the online platform, which may result in the under-sampling of young adults with lower socioeconomic status [65, 66] and sex differences in response behaviors (i.e., 64% of participants were female) [67]. For future study, researchers should explore utilizing more efficient techniques (such as multilevel regression and post-stratification) to address the representativeness of online surveys [68].

Forth, although the current study accounted for potential covariates when estimating the association between anxiety symptoms and meeting 24 h movement guidelines, it is possible that the association observed may be influenced by other factors (e.g., cardiometabolic health and mental health disorders) [25]. It would have been critical to explore the complete list of the potential confounding variables. However, the quality of current evidence was not satisfactory enough for researchers to do a thorough job of measuring and controlling for potential covariates [25]. Therefore, we call for more research on the associations, especially the longitudinal associations, between sociodemographic and health factors and 24 h movement behaviors.

Fifth, it is argued that measures of the SAS should be treated as a continuous variable, which can reduce the measurement errors in the relevant studies. However, the dichotomization of SAS is also sensible in clinical and medical applications because this can classify patients according to risks, provide clinical recommendations for additional treatments, and allocate medical resources according to patient need [69]. Although the SAS defines its dichotomy based on a reliable and valid threshold, dichotomization would result in information loss and a decrease in statistical power [70]. Whether dichotomy or continuity is a more effective method is beyond the scope of this research; so we do not discuss the issue in the current study. Some studies examine the degrees of anxiety symptoms using both categorical and continuous ratings [71], which implies that future studies can consider the use of continuous variables to further explain our research findings.

## Conclusion

The current study is one of the first to investigate the correlates of meeting 24 h movement guidelines and the relationship between anxiety symptoms and meeting the guidelines among Chinese university students. We observed that individuals who were male, had a better-educated mother, had more close friends, and perceived higher family affluence presented a higher probability of meeting the overall movement guidelines. Compared to meeting fewer recommendations from the 24 h movement guidelines, university students who met the overall guidelines showed the lowest incidence of anxiety symptoms. Following the recent release of the 24 h movement guidelines for adults, more longitudinal investigations should be conducted in heterogeneous adult populations to study adherence to the guidelines, their multidimensional correlates, and causal associations with health indicators.

## Data Availability

The datasets used and/or analyzed during the current study are available from the corresponding author on reasonable request.

## Abbreviations

SB: Sedentary Behavior
PA: Physical Activity
VIRTUE: Framework for Viable Integrative Research in Time-Use Epidemiology
IPAQ-SF: Physical Activity Questionnaire-Short Form
PSQI: Pittsburgh Sleep Quality Index
SAS: Self-rating Anxiety Scale
BMI: Body Mass Index
SD: Standard Deviation
OR: Odds Ratios
CI: Confidence Interval
